# GWAS identifies an ortholog of the rice D11 gene as a candidate gene for grain size in an international collection of hexaploid wheat

**DOI:** 10.1038/s41598-021-98626-0

**Published:** 2021-09-30

**Authors:** Honoré Tekeu, Eddy L. M. Ngonkeu, Sébastien Bélanger, Pierre F. Djocgoué, Amina Abed, Davoud Torkamaneh, Brian Boyle, Patrick M. Tsimi, Wuletaw Tadesse, Martine Jean, François Belzile

**Affiliations:** 1 Département de Phytologie, Université Laval, Quebec City, QC Canada; 2 Institut de Biologie Intégrative et des Systèmes, Université Laval, Quebec City, QC Canada; 3 Donald Danforth Plant Science Center, St. Louis, MO USA; 4 Institute of Agricultural Research for Development, Yaoundé, Cameroon; 5 Department of Plant Biology, University of Yaoundé I, Yaoundé, Cameroon; 6 Department of Plant Agriculture, University of Guelph, Guelph, ON Canada; 7 International Center for Agricultural Research in the Dry Areas (ICARDA), Beirut, Lebanon

**Keywords:** Genetics, Plant sciences, Biotechnology, Genomics

## Abstract

Grain size is a key agronomic trait that contributes to grain yield in hexaploid wheat. Grain length and width were evaluated in an international collection of 157 wheat accessions. These accessions were genetically characterized using a genotyping-by-sequencing (GBS) protocol that produced 73,784 single nucleotide polymorphism (SNP) markers. GBS-derived genotype calls obtained on Chinese Spring proved extremely accurate when compared to the reference (> 99.9%) and showed > 95% agreement with calls made at SNP loci shared with the 90 K SNP array on a subset of 71 Canadian wheat accessions for which both types of data were available. This indicates that GBS can yield a large amount of highly accurate SNP data in hexaploid wheat. The genetic diversity analysis performed using this set of SNP markers revealed the presence of six distinct groups within this collection. A GWAS was conducted to uncover genomic regions controlling variation for grain length and width. In total, seven SNPs were found to be associated with one or both traits, identifying three quantitative trait loci (QTLs) located on chromosomes 1D, 2D and 4A. In the vicinity of the peak SNP on chromosome 2D, we found a promising candidate gene (TraesCS2D01G331100), whose rice ortholog (D11) had previously been reported to be involved in the regulation of grain size. These markers will be useful in breeding for enhanced wheat productivity.

## Introduction

The grain size, which is associated with yield and milling quality, is one of the essential traits that have been subject to selection during domestication and breeding in hexaploid wheat^1^. During the domestication process from ancestral (Einkorn) to common wheat (*Triticum aestivum* L.) going through tetraploid species, wheat abruptly changed, from a grain with greater variability in size and shape to grain with higher width and lower length^2,3^. However, grain yield is determined by two components namely, the number of grains per square meter and grain weight. Following, grain weight is estimated by grain length, width, and area, which are components showing higher heritability than mainly yield in wheat^4^.

Larger grains may have a positive effect on seedling vigor and contribute to increased yield^5^. Geometric models have indicated that changes in grain size and shape could result in increases in flour yield of up to 5%^6^. Consequently, quantitative trait loci (QTLs) or genes governing grain shape and size are of interest for domestication and breeding purposes^7,8^. Many genetic mapping studies have reported QTLs for grain size and shape in wheat cultivars^1,2,8–10^ and some studies have revealed that the D genome of common wheat, derived from *Aegilops tauschii*, contains important traits of interest for wheat breeding^11,12^.

At the genomic level, Okamoto et al.^13^ performed QTL analyses for grain size and shape-related traits using four synthetic wheat F2 populations to identify the genetic loci responsible for grain size and shape variation in hexaploid wheat and found QTLs for grain length and width on chromosomes 1D and 2D. This is particularly interesting as the tenacious glume gene Tg-D1 on chromosome 2D is a well-known locus that has been recruited for the domestication of wheat grain size and shape. During allohexaploid wheat speciation, a dramatic change in grain shape occurred due to a mutation in the Tg-D1 gene^14^. Furthermore, Yan et al.^15^ reported a genomic region associated with grain size on chromosome 2D.

New advances in genomics technologies has revolutionized research in plants by developing new high throughput genotyping methods to increase knowledge of the genetic basis of diversity in large core collection of genetic materials through genome-wide association studies (GWAS). Based on such high-density SNP markers, GWAS can be used for the description and high-resolution mapping of genetic variance from collections of genetic ressources that have derived from several historical recombination cycles^16^. Furthermore, Genotyping-by-sequencing (GBS) is a Next-Generation Sequencing (NGS) technology for high-throughput and cost-effective genotyping, that provides a great potential for applying GWAS to reveal the genetic bases of agronomic traits in wheat^17^. Arora et al.^18^ conducted GWAS in a collection of *Ae. tauschii* accessions for grain traits, using SNP markers based on GBS. They identified a total of 17 SNPs associated with granulometric characteristics distributed over all seven chromosomes, with chromosomes 2D, 5D, and 6D harboring the most important marker-trait associations. On the other hand, most studies on germplasm of hexaploid wheat have focused on understanding the genetic and morphological diversity of this species. No studies have used GWAS based on GBS for economically important and essential grain yield components traits such as grain length and width in an international collection of hexaploid wheat. The present investigation aimed to identify QTLs and candidate genes governing grain length and width in an international collection of hexaploid wheat using a GBS-GWAS approach.

## Results

### Phenotypic characterization of grain yield components

**Figure 1:**
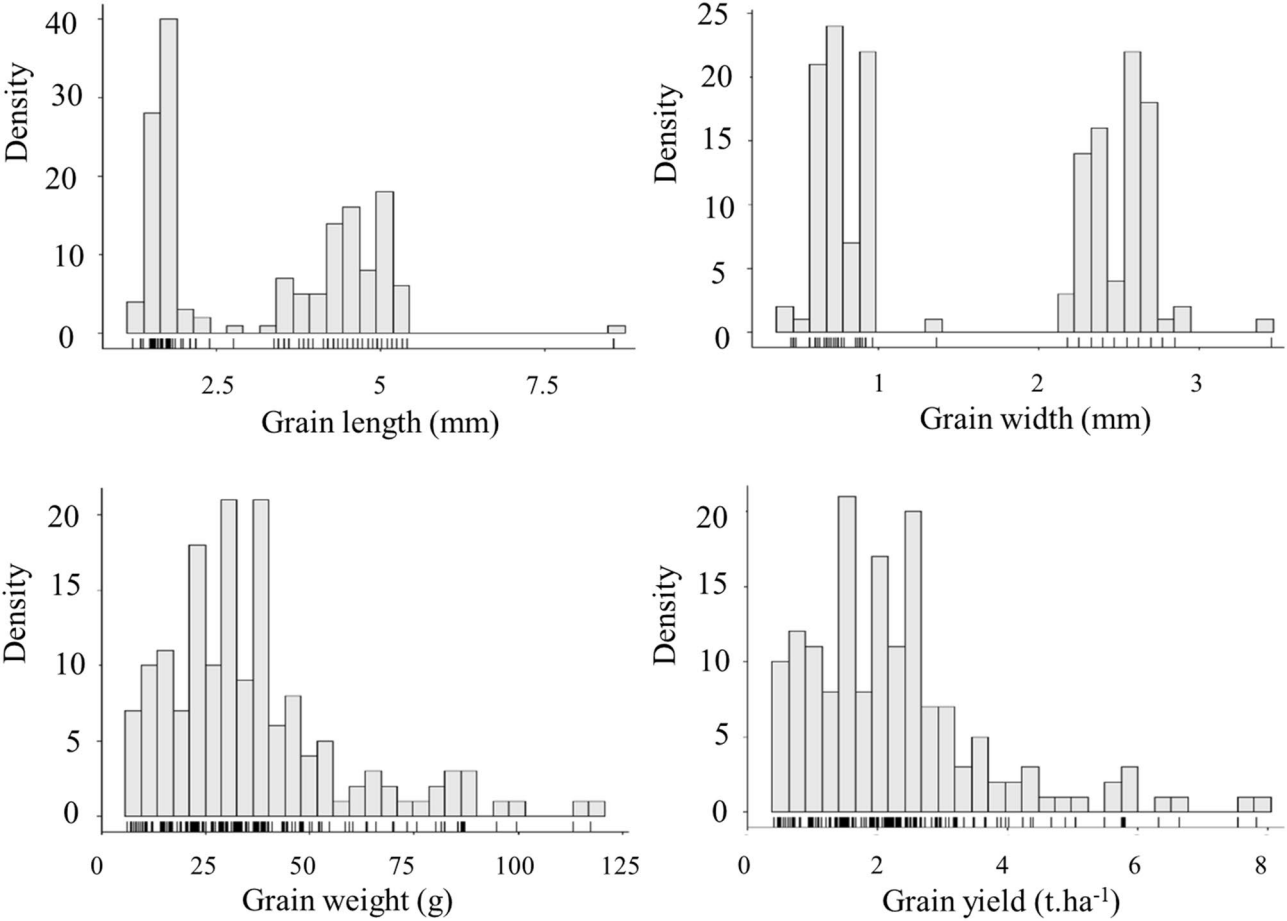
Distribution of phenotypes for grain length (upper left), grain width (upper right), grain weight (bottom left) and grain yield (bottom right). Histograms are based on the average trait value of each wheat line across the different environments. The bars under the histograms represent the density of individuals. Those phenotypes are referring only to the international panel of wheat and do not include the Canadian accessions.

To explore components of grain yield in wheat, we measured four phenotypes: grain length (Gle), grain width (Gwi), 1000-grain weight (Gwe) and grain yield (Gyi) over two years at two sites. Those phenotypes are referring only to the international panel of wheat and do not include the Canadian accessions. As shown in Table 1, means (± standard deviation) observed for these traits corresponded to: 3.28 mm (± 1.42) for grain length, 1.77 mm (± 0.88) for grain width, 36.17 g (± 21.77) for 1000-grain weight and 2.30 t/ha (± 1.44) for grain yield. The broad-sense heritability estimates were 90.6% for grain length, 97.9% for grain width, 61.6% for 1000-grain weight and 56.0% for grain yield. An analysis of variance revealed significant differences due to genotypes (G) for all traits and, for two traits (Gwe and Gyi), the interaction between genotype and environment (GxE) proved significant. A correlation analysis showed a high significant positive correlation between grain yield and grain weight (r = 0.94; *p* < 0.01) and also between grain length and grain width (r = 0.84; *p* < 0.01). Also, significant positive correlations were identified between grain yield and grain length (r = 0.50; *p* < 0.01) and between grain yield and grain width (r = 0.43; *p* < 0.01). Interestingly, a bimodal distribution was observed for grain length and width (Fig. 1). Together, these results suggest that a major gene controls two important characters related to grain size with a high heritability within this collection.

**Table 1:** Descriptive statistics, broad sense heritability (h^2^) and F-value of variance analysis for four agronomic traits in a collection of 157 wheat lines.

Traits	Unit	Range		Mean ± SD	h^2^	F-values		
		Min	Max			Genotype (G)	Environment (E)	G × E
Gle	mm	1.22	8.55	3.28 ± 1.42	90.6	10.7***	36.9	1.1
Gwi	mm	0.45	3.45	1.77 ± 0.88	97.9	48.6***	11.5	1.3
Gwe	g	6.25	117.38	36.17 ± 21.7	61.6	30.9***	15.7**	2.6*
Gyi	t/ha	0.42	7.83	2.30 ± 1.44	56.0	66.3***	174.9***	2.2*

*SD* Standard deviation, *h*^*2*^ Broad sense heritability, *Gle* Grain length, *Gwi* Grain width, *Gwe* 1000-grain weight, *Gyi* Grain yield.

***, ** and *: significant at *p* < 0.001, *p* < 0.01, and *p* < 0.05, respectively.

In examining the relationship between 1000-grain weight and grain length/width using bagplots on the collection of 159 accessions, no outliers were found when considering the relationship between grain weight and width. In contrast, two accessions (Attila3, Babax8) were indeed detected as outliers when comparing grain weight and length (Supplementary Fig. S1). In the later steps (analysis of population structure and GWAS) we excluded these two accessions considered to be outliers.

### Genome-wide SNP marker discovery and validation

To genetically characterize our wheat collection and study the genetic determinants of grain size, we used a double digestion (PstI/MspI) GBS approach to genotype this collection. Overall, 77,124 and 73,784 SNPs were discovered for the set of 71 Canadian wheat accessions and 157 exotics wheat accessions, respectively.

To assess the reproducibility and accuracy of genotypes called via the GBS approach, we genotyped 12 different plants of CS (i.e. biological replicates), which were added to the set of 288 wheat samples for SNP calling and bioinformatics analysis. Sequence reads of the full set of 300 wheat samples obtained from GBS were analyzed following the standard steps of SNP calling and bioinformatics analysis described below. This yielded a total of 129,940 loci that were used for the assessment of accuracy and reproducibility of SNP calls. For each individual plant of CS, the GBS calls were compared between replicates and with the Chinese Spring reference genome (at the corresponding positions).

On the non-imputed data, we detected a very high level of concordance (99.9%) between the genotypes of each CS individual and the reference alleles for the 1,196,184 called genotypes ([130 K SNPs × 12 samples]—missing data; Supplementary Fig. S2). Among those 12 biological replicates of CS, we found a very high reproducibility of genotype calls, as the pairwise identity of genetic distance calls varied from 1.56E−04 to 5.08E−04, with an average of 2.86E−04. In order to ensure about identity of each CS plant, we have found that this value between the individual w56_Guelph (Canadian wheat variety) and each of the CS plant is greater than 0.1.

After imputation of the missing genotype calls, we observed a mean concordance of 93.8% between the CS individuals and the CS reference genome. Furthermore, 76.7% of genotypes were called initially and 23.3% of genotypes were imputed. It should be noted that the accuracy rate for imputing missing data is 73.4%. More details of SNP data set are provided in supplementary Table S1.

**Figure 2:**
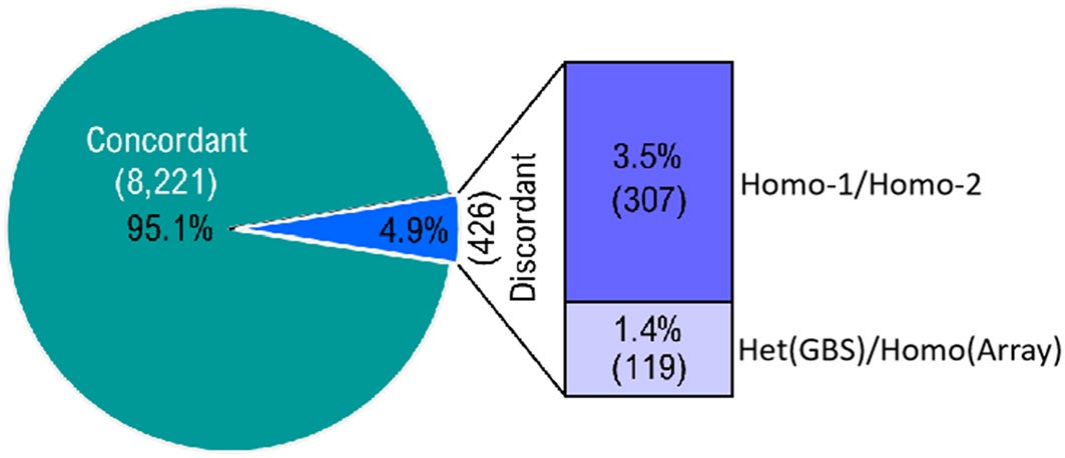
Concordance of genotype calls made using both marker platforms (GBS and 90 K SNP Array). GBS-derived SNP genotypes were compared to the genotypes called at loci in common with the 90 K SNP Array for the same 71 wheat samples.

As a further examination of data quality, we compared the genotypes called using both GBS and a SNP array on a subset of 71 Canadian wheat accessions that had been previously genotyped using the 90 K SNP array. A total of 77,124 GBS-derived and 51,649 array-derived SNPs were discovered in these 71 accessions (Supplementary Table S2). Of these, only 135 SNP loci were common to both platforms and among these potential 9,585 datapoints (135 loci × 77 lines), only 8,647 genotypes could be compared because the remaining 938 genotypes were missing in the array-derived data. As shown in Fig. 2, a high level of concordance (95.1%) was seen between genotypes called by both genotyping approaches. To better understand the origin of discordant genotypes (4.9%), we inspected the set of 429 discordant SNP calls and observed that: (1) 3.5% of discordant calls corresponded to homozygous calls of the opposite allele by the two technologies; and (2) 1.4% of discordant calls were genotyped as heterozygous by GBS while they were scored as homozygous using the 90 K SNP array. More details are provided in Supplementary Table S3. From these comparisons, we conclude that GBS is a highly reproducible and accurate approach for genotyping in wheat and can yield a greater number of informative markers than the 90 K array.

### Genome coverage and population structure

For the full set of accessions, a total of 129,940 SNPs was distributed over the entire hexaploid wheat genome. The majority of SNPs were located in the B (61,844) and A (50,106) sub-genomes compared to the D (only 17,990 SNPs) sub-genome (Table 2). Although the number of SNPs varied two to threefold from one chromosome to another within a sub-genome, a similar proportion of SNPs was observed for the same chromosome across sub-genomes. Typically, around half of the markers were contributed by the B sub-genome (47.59%), 38.56% by the A sub-genome and only 13.84% by the D sub-genome.

**Table 2:** Distribution of SNP markers across the A, B and D genomes.

Chromosomes	Wheat genome			Total
	A (*)	B (*)	D (*)	
1	6099 (0.36)	8115 (0.48)	2607 (0.15)	16,821 (0.13)
2	8111 (0.35)	11,167 (0.48)	3820 (0.17)	23,098 (0.18)
3	6683 (0.33)	10,555 (0.53)	2759 (0.14)	19,997 (0.15)
4	6741 (0.58)	4007 (0.34)	913 (0.08)	11,661 (0.09)
5	6048 (0.38)	8015 (0.51)	1719 (0.11)	15,782 (0.12)
6	5995 (0.33)	10,040 (0.55)	2191 (0.12)	18,226 (0.14)
7	10,429 (0.43)	9945 (0.41)	3981 (0.16)	24,355 (0.19)
Total	50,106	61,844	17,990	129,940

*Proportion of markers on a homoeologous group of chromosomes that were contributed by a single sub-genome.

**Figure 3:**
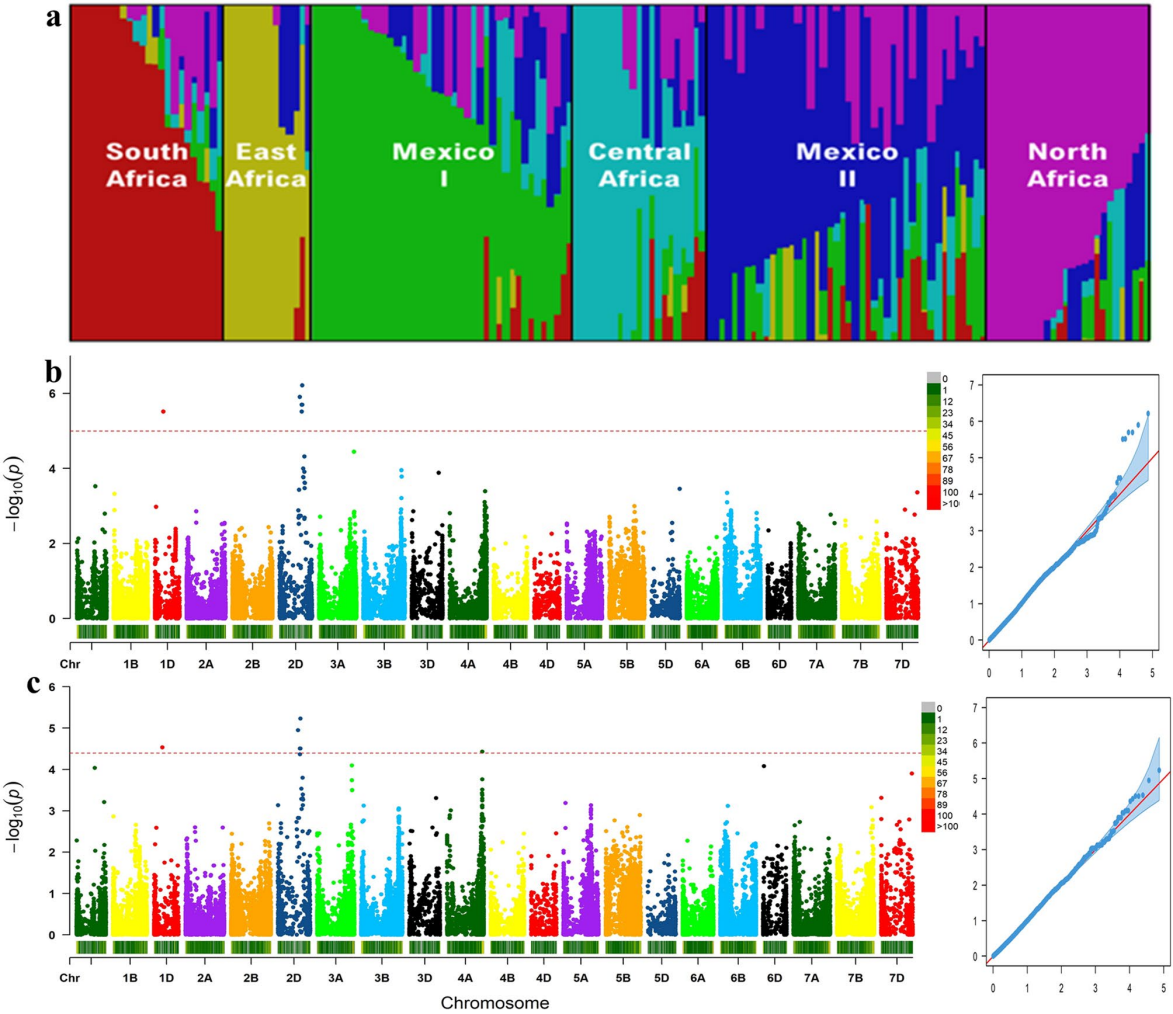
Population structure of 157 hexaploid wheat cultivars and genome-wide association studies of grain traits (**a**). Manhattan and Q–Q plots indicate the degree of association between SNPs and grain length (**b**) or grain width (**c**). Population structure plot and Manhattan/Q-Q plots were generated using fastSTRUCTURE version 1.0 (https://rajanil.github.io/fastStructure/) and GAPIT version 2 (https://pubmed.ncbi.nlm.nih.gov/27898829/), respectively.

The analysis of population structure for the accessions of the association panel showed that K = 6 best captured population structure within this set of accessions and these clusters largely reflected the country of origin (Fig. 3). The number of wheat accessions in each of the six subpopulations ranged from 6 to 43. The largest number of accessions was found in northwestern Baja California (Mexico) represented here by Mexico 1 (43) and the smallest was observed in East and Central Africa (6).

### GWAS analysis for marker-trait associations for grain size

To identify genomic loci contributing to grain size in wheat, we performed a GWAS analysis on 157 accessions (excluding the two accessions considered to be outliers) and 73,784 SNPs. As seen in Fig. 3, both Q–Q plots suggest that the confounding effects of population structure and relatedness were well controlled. For both traits, the greatest marker-trait associations were detected at the end of chromosome 2D, while another weaker association was shared at the beginning of chromosome 1D. For grain width only, a marker-trait association was detected on chromosome 4A. In total, seven SNPs were found to be associated with one or both traits, with respectively one, five and one significant SNPs being located on chromosomes 1D, 2D and 4A. Except for two SNPs (chr2D:442798939 and chr4A:713365388), all other SNPs were significant for both grain length and grain width. The SNP at 4A:713365388 was significant only for grain width while the SNP at 2D:442798939 was significant only for grain length.

The most significant association was observed on chromosome 2D and contributed to both grain length and grain width (Table 3, Fig. 3). For this QTL, a total of four SNPs was observed and the SNP most significantly associated to both traits was located at position 2D:452812899. A fifth SNP located at 2D:442798939 was significantly associated to grain length only, but was just below the significance threshold (*p*-value = 4.34E−05) for grain width.

**Table 3:** Details of loci associated with grain size traits identified via a genome-wide association study in a collection of 157 hexaploid wheat lines.

Loci	Chr	Grain traits	*P* value	MAF	R^2^	Allelic effect	Alleles
*chr1D:166874041*	1D	Length	3.07E−06	0.30	0.11	0.76	T/C
		Width	2.94E−05	0.30	0.06	0.33	
*chr2D:403935865*	2D	Length	1.25E−06	0.29	0.12	0.79	T/C
		Width	1.12E−05	0.29	0.07	0.34	
*chr2D:442798939*	2D	Length	3.07E−06	0.29	0.11	− 0.77	A/G
*chr2D:444560418*	2D	Length	2.02E−06	0.28	0.11	− 0.80	A/G
		Width	3.12E−05	0.28	0.06	− 0.34	
*chr2D:452644656*	2D	Length	2.02E−06	0.28	0.11	− 0.80	A/G
		Width	3.12E−05	0.28	0.06	− 0.34	
*chr2D:452812899*	2D	Length	6.15E−07	0.31	0.13	− 0.81	A/G
		Width	5.89E−06	0.31	0.07	− 0.35	
*chr4A:713365388*	4A	Width	3.74E−05	0.14	0.06	0.36	A/G

*Chr* Chromosome, *MAF* Minor allele frequency, *R*^*2*^ R square of model with SNP, calculated by *R*^2^ of model with SNP minus *R*^2^ of model without SNP^48^.

A high degree of LD was detected among some of the seven SNPs from chromosome 2D displaying association with grain traits. These formed one discontinuous linkage block as the LD between markers belonging to this block was higher (mean of r^2^ = 0.90). For this reason, we considered these to define one quantitative trait locus (QTL) on chromosome 2D (Supplementary Fig. S3). This QTL included 5 SNP markers (chr2D:403935865, chr2D:442798939, chr2D:444560418, chr2D:452644656 and chr2D:452812899) and the peak SNP (chr2D:452812899) explained between 7 and 13% of the phenotypic variation for grain length and width. The minor allele frequency (MAF) at this locus was 0.31 and exerted an allelic effect from − 0.81 to − 0.35 mm (Table 3).

On chromosome 1D, the SNP marker chr1D:166874041 defined a QTL for both grain length and width. The percentage of phenotypic variation explained by this marker for grain length and width was 11% and 6% respectively, with a MAF of 0.30 and allelic effects of 0.76 and 0.33 mm for grain length and width, respectively. Furthermore, a high degree of interchromosomal LD was observed among the peak SNPs between chromosomes 1D and 2D (r^2^ = 0.94) displaying association with grain traits. In addition, almost all accessions which have the major allele on chromosome 1D are the same which have the major allele on chromosome 2D. Thus, the combined impact of these two loci could explain the observed bimodal distribution.

On chromosome 4A, the SNP marker chr4A:713365388 defined a QTL for grain width only and it explained 6% of the variation, had a MAF of 0.14 and exerted an allelic effect of 0.36 mm. However, we reported a very weak LD between this peak SNP marker and the two others on chromosomes 1D and 2D.

In summary, a total of three QTLs significantly associated with grain length and/or width were identified on chromosomes 1D, 2D and 4A.

### Candidate gene detection for grain size

**Figure 4:**
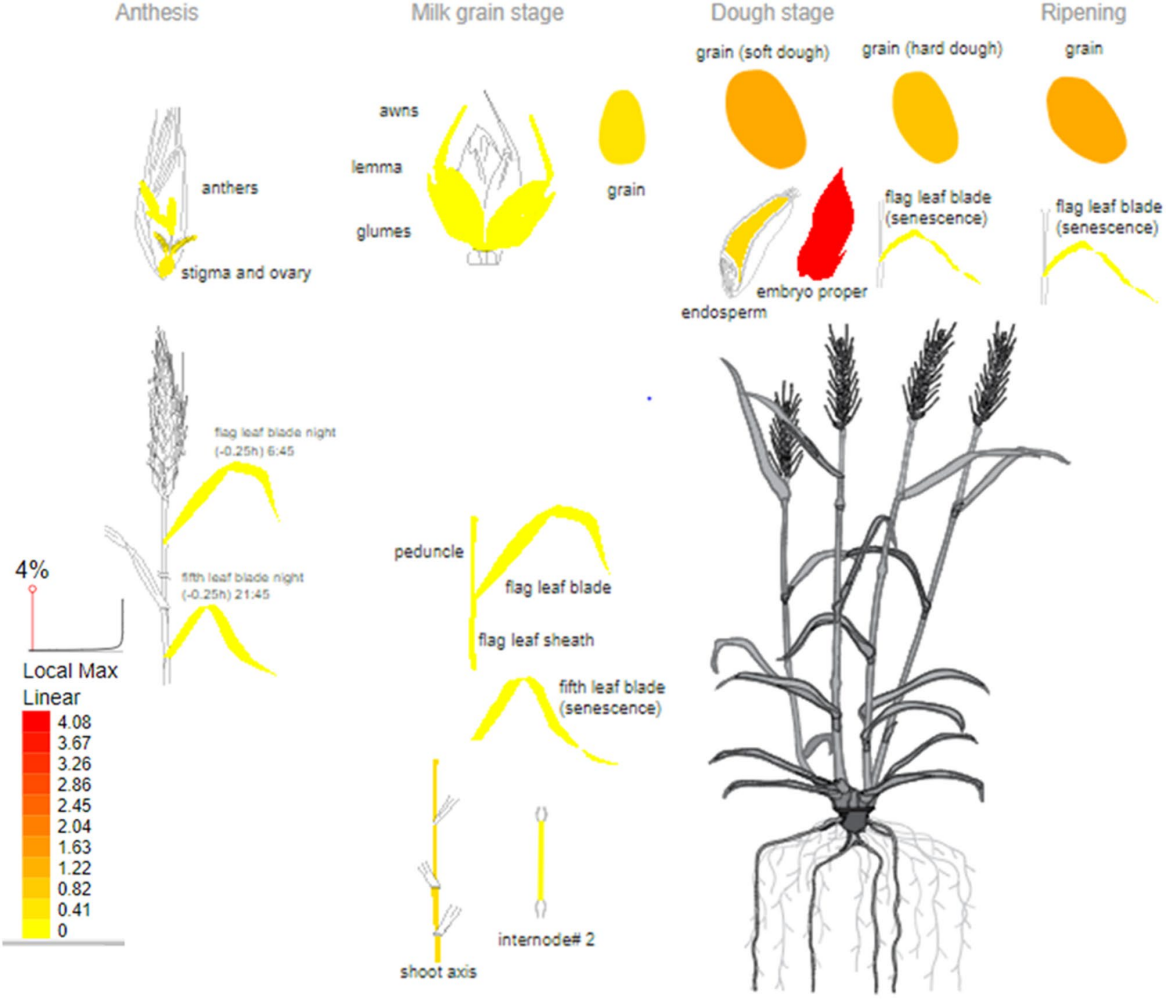
Expression profile of TraesCS2D01G331100 gene based on transcriptomic analysis in wheat. As shown, this gene is most highly expressed in the developing embryo during embryogenesis and grain development in wheat. Data for this view derived from RNA-seq of wheat^48^ and the image was generated with the eFP (RNA-Seq data) at http://bar.utoronto.ca/eplant/ by Waese et al.^51^. The legend at bottom left presents the expression levels, coded by colors (yellow = low, red = high).

To identify candidate genes contributing to grain size within the studied wheat collection, we investigated the genes residing in the same linkage block as the peak SNP for each QTL. On chromosome 2D, the QTL with the largest number of associated SNPs (chr2D:403935865 to chr2D:452811303) included a total of 315 high-confidence genes of which 66 genes are expressed during embryogenesis and grain development in wheat. On chromosomes 1D and 4A, the linkage blocks harboring SNP markers chr1D:166874041 and chr4A:713365388, each defining a QTL, did not include high-confidence genes. Upon examination of the annotations and gene expression profile for the candidate genes, the most promising appears to be the TraesCS2D01G331100 gene in the QTL on chromosome 2D, which is most highly expressed in the developing embryo during embryogenesis and grain development in wheat (Fig. 4). As well, it is expressed in both endosperm and pericarp, and was found to encode a cytochrome P450 (CYP724B1), which showed homology to enzymes involved in brassinosteroid biosynthesis, indicating the mechanism by which seed size may be regulated in wheat. It is an ortholog of the rice CYP724B1 gene, commonly known as the D11 gene. The D11 gene was previously reported as being involved in the regulation of internode elongation and seed development due to its role in the synthesis of brassinosteroids, key regulators of plant growth promoting the expansion and elongation of cells. More details are provided in Supplementary Table S4.

### Haplotypes at the wheat orthologue of the rice D11 gene and their phenotypic effects

**Figure 5:**
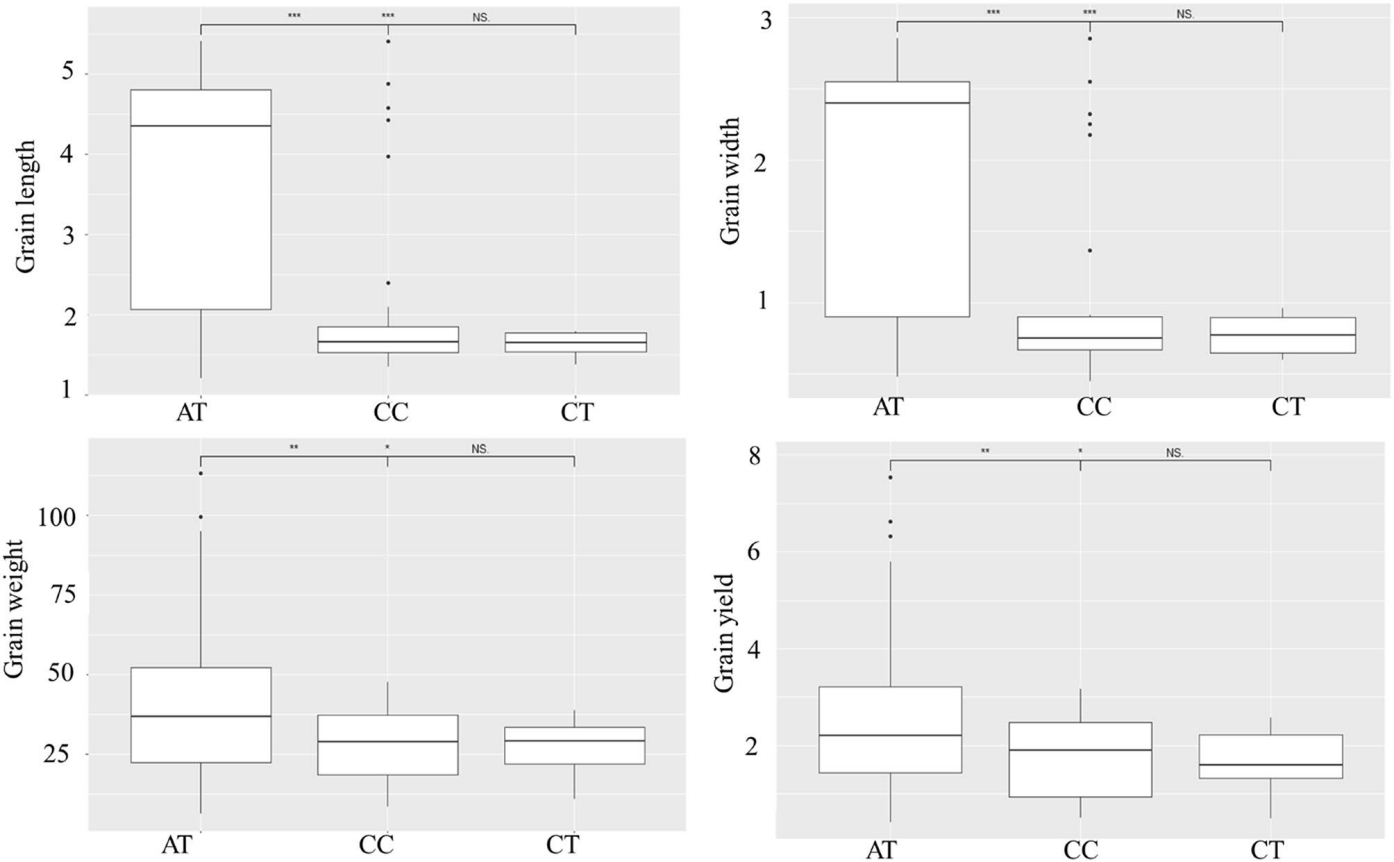
Impact of haplotypes on the grain traits and yield (using Wilcoxon test). Boxplots for the grain length (upper left), grain width (upper right), grain weight (bottom left) and grain yield (bottom right) are represented for each haplotype. ***, ** and *: significant at *p* < 0.001, *p* < 0.01, and *p* < 0.05, respectively. *NS* Not significant.

To provide a useful breeding tool for the main QTL identified in this research, we defined SNP haplotypes around our candidate gene. Using HaplotypeMiner, we identified two SNPs (chr2D:423365752 and chr2D:425474599, Supplementary Fig. S4) that best captured the SNP landscape in the vicinity of the candidate gene. These markers reside in the same haplotype block as the SNP markers, but were not individually found to be significantly associated with grain width and length. These SNP markers define three haplotypes (AT, CT or CC) around the candidate gene, with 99, 18 and 40 individuals carrying these haplotypes, respectively. To investigate the phenotypes associated with these haplotypes, we analyzed the trait value for each haplotype. Interestingly, we observed that for all traits, the mean values of accessions with haplotype AT were significantly larger (*p* < 0.001) than those obtained for the other haplotypes. As shown in Fig. 5, accessions carrying haplotype AT showed mean values of 3.76 mm for grain length, 2.02 mm for grain width, 40.87 g for grain weight and 2.55 t/ha for grain yield, compared to 2.16 mm, 1.05 mm, 26.87 g and 1.75 t/ha (respectively for grain length, width, weight and yield) for accessions carrying haplotype CC and 1.65 mm, 0.78 mm, 26.89 g and 1.69 t/ha (respectively for grain length, width, weight and yield) for accessions carrying haplotype CT. Furthermore, the relation between the 3 haplotypes and the 6 groups found in the population analysis showed that the haplotype AT predominates in the populations of Mexico 1 and North Africa (Supplementary Fig. S5, Supplementary Table S5). To conclude, we suggest that SNP markers corresponding to haplotype AT will provide a useful tool in marker-assisted breeding programs to improve wheat productivity. Therefore, we point out that the relationship between yield and haplotypes around the D11 gene would allow the selection of high-yielding wheat lines in a breeding program.

## Discussion

The goal of our study was to identify genomic regions controlling variation for grain size in an international collection of 157 hexaploid wheat accessions through a GWAS approach. Thus, we collected the phenotypes for three grain traits (length, width, weight) in addition to grain yield. A statistical analysis revealed that the genotype was a major source of variance for all traits and that these exhibited a high heritability. In agreement with Arora et al.^18^ in *Ae. tauschii* and Rasheed et al.^19^ in wheat, we observed that grain length, grain width and grain weight were positively correlated to grain yield. Interestingly, a bimodal distribution was observed for both the grain length and width phenotypes, suggesting that one to a few major genes control these traits in our collection.

To assess the reproducibility and accuracy of genotypes called via the GBS approach, we genotyped 12 different plants of Chinese Spring (i.e. biological replicates), which were added to the set of 288 wheat samples for SNP calling and bioinformatics analysis, which yielded a total of 129,940 loci. Among the 12 biological replicates of CS, we found a very high reproducibility (~ 100%) in our genotype calls. Firstly, we verified the quality of our SNP data by investigating the reproducibility and accuracy of GBS-derived SNPs calls, and found that GBS-derived genotypes were in agreement with the reference genome in 99.9% of cases in over 1 M comparisons for non-imputed data and 93.8% after imputation of the missing genotype calls. Recently, Abed et Belzile^20^ reported that the accuracy of SNP calls was 99% for non-imputed and 89% for imputed SNPs dataset in Barley. In our study, 76.7% of genotypes were called initially, and only 23.3% were imputed. Thus, we conclude that the imputed data are of lower reliability.

As a further examination of data quality, we compared the genotypes called by GBS and a 90 K SNP array on a subset of 71 Canadian wheat accessions. Among the 9,585 calls available for comparison, 95.1% of calls were in agreement. It is likely that both genotyping methods contributed to cases of discordance. It is known, however, that the calling of SNPs using the 90 K array is challenging because of the presence of three genomes in wheat and the fact that most SNPs on this array are located in genic regions that tend to be typically more highly conserved, thus allowing for hybridization of homoeologous sequences to the same element on the array^21,22^. The fact that the vast majority of GBS-derived SNPs are located in non-coding regions makes it easier to distinguish between homoeologues^21^. This likely contributed to the very high accuracy of GBS-derived calls described above. We conclude that GBS can yield genotypic data that are at least as good as those derived from the 90 K SNP array. This is consistent with the findings of Elbasyoni et al.^23^ as these authors concluded that “GBS-scored SNPs are comparable to or better than array-scored SNPs” in wheat genotyping. Likewise, Chu et al.^24^ observed an ascertainment bias for wheat caused by array-based SNP markers, which was not the case with GBS.

Confident that the GBS-derived SNPs provided high-quality genotypic information, we performed a GWAS to identify which genomic regions control grain size traits. A total of three QTLs located on chromosomes 1D, 2D and 4A were discovered. Under these QTLs, seven SNPs were found to be significantly associated with grain length and/or grain width. Five SNPs were associated to both traits and two SNPs were associated to one of these traits. The QTL located on chromosome 2D shows a maximum association with both traits. Interestingly, previous studies have reported that the sub-genome D, originating from *Ae. tauschii*, was the main source of genetic variability for grain size traits in hexaploid wheat^11,12^. This is also consistent with the findings of Yan et al.^15^ who performed QTL mapping in a biparental population and identified a major QTL for grain length that overlaps with the one reported here. In a recent GWAS on a collection of *Ae. tauschii* accessions, Arora et al.^18^ reported a QTL on chromosome 2DS for grain length and width, but it was located in a different chromosomal region than the one we report here. With a view to develop useful breeding markers to improve grain yield in wheat, SNP markers associated to QTL located on chromosome 2D appear as the most promising.

It is worth noting, however, that another genomic region of interest was also located on the D subgenome. Interestingly, the peak SNP on 1D exhibited a very high degree of LD with the peak SNP on 2D. This may reflect that, when selecting for large seed size, favorable alleles at both QTLs tend to be captured. In biparental progeny segregating for both loci, it would be interesting to assess if there are any epistatic effects between these QTLs leading to both loci being required to achieve the full phenotypic effect.

To identify a candidate gene contributing to grain length and width, we examined the genes residing in the same linkage block as the peak SNP for each QTL. In the genomic interval spanned by the QTL contributing the most to the phenotypic variation for grain size (2D_40.4–45.1 Mb), a total of 66 high-confidence genes expressed during embryogenesis and grain development were observed. The *TraesCS2D01G331100* gene seems like a highly promising candidate as it is most highly expressed in the developing embryo during embryogenesis and grain development in wheat. As well, it is expressed at the corresponding endosperm and pericarp, and was found to encode the cytochrome P450 (CYP724B1), which showed homology to enzymes involved in brassinosteroid biosynthesis, indicating the mechanism by which grain size is regulated in wheat. Furthermore, this gene has been well conserved during the domestication process from ancestral (Einkorn) to common wheat (*Triticum aestivum* L.) going through tetraploid species^25^. It is an orthologous to the rice *CYP724B1* gene, commonly known as the *D11* gene. The latter has been reported as involved in the regulation of internode elongation and seed development due to his role in brassinosteroid synthesis^26^. Brassinosteroids are a group of plant hormones and are key regulators of plant growth and development (including seeds) that promote cell expansion and elongation^27^.

To further refine the association between the *TraesCS2D01G331100* gene and grain width and length, we defined SNP haplotypes. An analysis of haplotypes surrounding this gene identified three distinct haplotypes, and we observed that, for all grain size traits, the phenotypes corresponding to haplotype AT displayed significantly higher values than those of other haplotypes. We therefore suggest that SNP markers flanking *TraesCS2D01G331100* could provide a useful tool in marker-assisted breeding programs to improve wheat productivity by selecting alleles leading to larger grain size and higher yield. In the longer term, it would be interesting to define more precisely the exact nature of the alleles at this gene through targeted re-sequencing of this gene in a broader collection of accessions.

## Materials and methods

### Plant materials and phenotyping

A total of 228 hexaploid wheat (*Triticum aestivum* L.) varieties were used in our study. These accessions comprised two groups. A first group of 71 Canadian accessions was used to validate the accuracy of GBS in wheat. The second group of 157 accessions was used for genome-wide association analyses. Indeed, accessions were collected from many wheat breeding programs. Canadian accessions were collected from the University of Guelph Wheat Breeding Program and accessions from the second group were collected from South Africa through the Agricultural Research Council (ARC), Stellenbosch University’s Plant Breeding Laboratory (SU-PBL) and SENSAKO’s breeding programs, East Africa and Mexico via the International Maize and Wheat Improvement Center (CIMMYT), Central Africa by the Institute of Agricultural Research for Development (IRAD) and from farmers^28^, and North Africa per the International Center for Agricultural Research in the Dry Areas (ICARDA). With the latter accessions, field trials were conducted in two different trial sites in the bimodal humid forest zone of Cameroon, during the 2015–2016 wheat-growing seasons in Mbankolo (1057 m above sea level) and during 2016–2017 in Nkolbisson (650 m a. s. l.). In Mbankolo, the average temperature is 18–20 °C, bimodal rainfall with an annual average of 1600 mm. In Nkolbisson, the annual average temperature is 23.5 °C, the rainfall is bimodal with an annual average of 1560 mm. At each trial site, an incomplete alpha-lattice design with two replications was used. Each accession was planted in five-row plots, in 3-m rows with 5 cm between plants and 25 cm between rows. Then, fields trials were managed in accordance with the technical recommendations and standard agricultural practices for wheat^29^. Grain length (Gle), grain width (Gwi), 1000-grain weight (Gwe) and grain yield (Gyi) were recorded for each accession. Gle and Gwi were measured by a digital Vernier caliper on 20 seeds per variety randomly picked from a pool of grains from each harvested area^18^.

### Analysis of phenotypic data

The analysis of variance for each trait was performed using PROC MIXED in SAS 9.4. Each cultivar was considered as a fixed effect, whereas replications and environments were considered as random effects. Pearson correlation coefficients between pairs of phenotypic traits were computed using Pearson’s correlation in SPSS 20.0. We estimated the broad-sense heritability (h^2^) for each trait using the following formula: $h^{2} = \frac{{V_{G} }}{{V_{G} + V_{GE} + V_{e} }}$, where V_G_: genetic variance; V_GE_: genetic × environment variance and V_e_: error variance.

### DNA isolation, GBS library construction and sequencing

Genomic DNA was extracted from dried young leaf tissue (~ 5 mg) for all accessions using a CTAB DNA isolation method^30^. Then, DNA was quantified using a Quant-iT™ PicoGreen (ThermoFisher Scientific, Canada) and the concentrations were normalized to 20 ng/μl for library preparation. Our 228 DNA samples were part of a larger set of 288 wheat samples on which GBS analysis was performed simultaneously (Fig. 5). In brief, 96-plex *Pst*I-*Msp*I GBS libraries were constructed^20,31,32^ and each was sequenced on three PI chips on an Ion Proton sequencer at the Plate-forme d’Analyses Génomiques of the Institut de Biologie Intégrative et des Systèmes (Université Laval, Québec, Canada). To allow an assessment of the quality of GBS-derived SNP calls, 12 independent samples of Chinese Spring (CS) DNA (each from a different plant) were used to produce a single (12-plex) *Pst*I/*Msp*I library that was sequenced on one PI chip.

### Single nucleotide polymorphism calling and bioinformatics analysis

**Figure 6:**
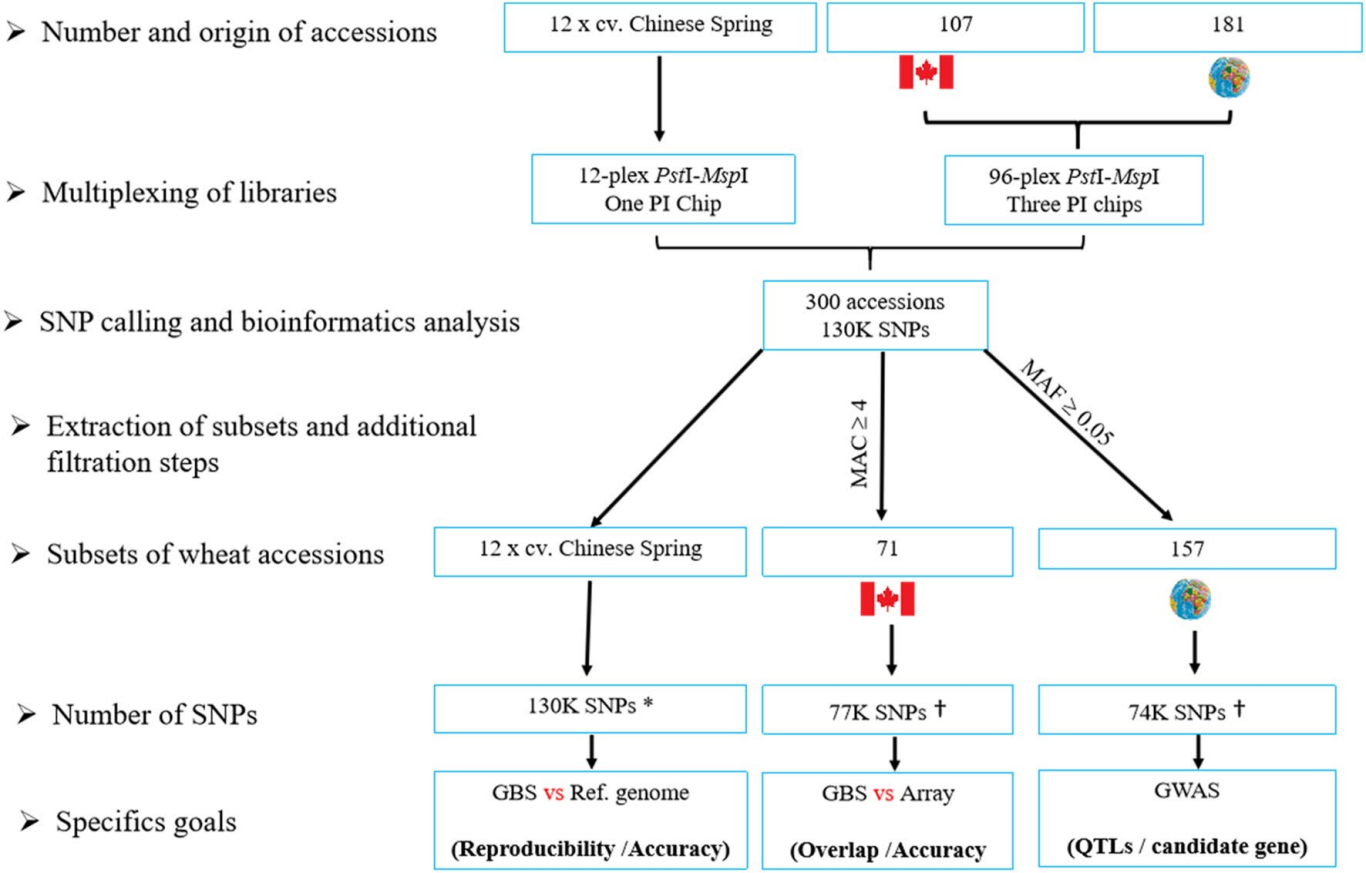
Schematic representation of the genetics analytical steps of wheat accessions subset. *vast majority of these are polymorphisms between Chinese Spring and the other accessions; † these are SNPs that are polymorphic within the accessions of these sub-collections. *MAC* Minor allele count, *MAF* Minor allele frequency.

DNA sequences of the full set (n = 300) of wheat samples obtained from GBS were analyzed using the Fast-GBS pipeline^33^ to align reads on the wheat reference genome (Chinese Spring v1.0) and to call SNPs. Fast-GBS results were first filtered to (i) keep only SNPs having the label “PASS” and SNPs positioned on chromosomes (i.e. not on scaffolds), (ii) remove indels and multiallelic SNPs, (iii) convert all heterozygous calls with genotype quality (GQ) < 30 to missing data, (iv) keep only SNPs with a minor allele count (MAC) ≥ 4, (v) remove accessions with more than 80% of missing data, (vi) exclude SNPs with more than 10% heterozygotes and (vii) exclude SNPs with missing data (N) > 80%. Finally, missing data were imputed using BEAGLE v5^34^ with the parameters described in Torkamaneh and Belzile^35^. Imputed genotypes were also filtered to keep only SNPs with a minor allele count (MAC) ≥ 4. After these standard filtration steps, three subsets of accessions were extracted from the full dataset for specific goals: (1) establish the accuracy and reproducibility of GBS-derived SNP calls on 12 replicates of cv. Chinese Spring; (2) compare SNP genotypes obtained via GBS and the 90 K array on a subset of 71 Canadian accessions and (3) perform GWAS for grain size on a diversity panel of 157 accessions. Additional filtration steps were performed on these subsets before these analyses. The imputed genotypes of the subset of 71 wheat accessions were filtered to keep only SNPs with a minor allele count (MAC) ≥ 4 and exclude SNPs with more than 10% heterozygotes, while those from the collection of 157 wheat accessions were filtered to keep only SNPs with a minor allele frequency (MAF) ≥ 0.05 (Fig. 6).

### Validation of SNP call accuracy

The SNP genotypes for 12 different cv. Chinese Spring plants were used to assess the accuracy and reproducibility of GBS-derived SNP calls. Before and after imputation of missing data, we measured both the degree of agreement in SNP calls between replicates and the agreement between the GBS-derived SNP calls and the Chinese Spring reference genome V1.0 using an in-house script. To compare the accuracy of GBS-based and array-based genotype calls, we used a set of 71 Canadian wheat accessions for which genotypic data for 51,649 SNPs had been obtained previously using the 90 K SNP Infinium iSelect array^36^. For the 135 SNPs called in common using both methods, genotype calls were compared using an in-house script.

### Population structure and linkage disequilibrium analyses

An analysis of population structure was performed on the collection of 157 wheat accessions (excluding the two accessions considered to be outliers) using fastSTRUCTURE version 1.0^37^ on SNP markers filtered at MAF ≥ 0.05 as recommended by Sobota et al.^38^. Population structure was evaluated using the filtered set of SNP markers using a simple prior and 1,000 iterations for K ranging from 1 to 12. The optimal range of K was determined based on model complexity using the marginal likelihood method using the fastSTRUCTURE script chooseK.py, as well as on visualization of the log marginal likelihood, and population visualization using Distruct version 1.1^39^.

Genome-wide linkage disequilibrium (LD) analysis was performed using PLINK version 1.9^40^, via the Gabriel method^41^. This method is based on a confidence interval and a normalized measure of D′. The pattern and distribution of intrachromosomal LD were visualized with LD plots generated using Haploview version 4.2^42^ to investigate the average LD decay along chromosomes. The smoothed second-degree LOESS curve was fitted to determine the critical D′ and r^2^ between loci.

### Genome-wide association study for grain traits

GWAS for grain traits was performed on the subset of 157 wheat accessions via the Genomic Association and Prediction Integrated Tool (GAPIT) version 2^43^. This approach, based on associations between the estimated genotypic values (BLUEs) for each trait and individual SNP markers^44,46^ was conducted with a compressed mixed linear model^45^. A matrix of genomic relationships among individuals (Supplementary Fig. S6) was calculated using the Van Raden method^43^. The statistical model used was: *Y* = Xβ + Z*u* + **ε**, where *Y* is the vector of phenotypes; *β* is a vector of fixed effects, including single SNPs, population structure (Q), and the intercept; *u* is a vector of random effects including additive genetic effects as matrix of relatedness between individuals (the kinship matrix), *u* ~ N(0, **K***σ*_*a*_^2^), where *σ*_*a*_^2^ is the unknown additive genetic variance and *K* is the kinship matrix; **X** and **Z** are the design matrices of β and *u*, respectively; and ε is the vector of residuals, **ε** ~ N(0, *Iσ*_*e*_^2^), where *σ*_*e*_^2^ is the unknown residual variance and *I* is the identity matrix. Association analysis was performed while correcting for both population structure and relationships among individuals with a combination of either the Q + K matrices; K matrix was computed using the Van Raden method^43^. The *p* value threshold of significance of the genome-wide association was based on false discovery rate (FDR-adjusted *p* < 0.05).

### Identification of candidate genes for grain size

To identify candidate genes affecting grain size in wheat, we defined haplotype blocks containing the peak SNP. Each region was visually explored for its LD structure and for genes known to reside in such regions. The associated markers located in the same LD block as the peak SNP were searched and positioned on the wheat reference genome v1.0 on the *International Wheat Genome Sequencing Consortium* (IWGSC) website (https://urgi.versailles.inra.fr/jbrowseiwgsc/gmod_jbrowse), and the annotated genes within each interval were screened based on their confidence and functional annotation thanks to the annotated and ordered reference genome sequence in place by IWGSC et al.^47^. Candidate genes potentially involved in grain size traits were further investigated by analyzing gene structure and crossing-referenced them against genes reported as controlling grain size in other Triticeae as well as orthologous search in other grass species^15,18,25,48–50^. Furthermore, the selected genes were further evaluated for their likely function based on publicly available genomic annotation. The function of these genes was also inferred by a BLAST of their sequences to the UniProt reference protein database (http://www.uniprot.org/blast/). To further provide more information about potential candidate genes, we used RNA-seq data of Ramírez-González et al.^48^, based on the electronic fluorescent pictograph (eFP) at bar.utoronto.ca/eplant (by Waese et al.^51^) to identify in what tissues and at which developmental stages candidate genes were expressed in wheat.

### Identification of haplotypes around a candidate gene

To better define the possible alleles in a strong candidate gene, we used HaplotypeMiner^52^ to identify SNPs flanking the *TraesCS2D01G331100* gene. For each haplotype, we calculated the trait mean (grain length, width, weight and yield) for lines sharing the same haplotype using the R ggpubr program^53^.

### Ethics declarations

Experiments on wheat were carried out in accordance with national, international guidelines.

## Supplementary Information

Supplementary Figures.

Supplementary Table S1.

Supplementary Table S2.

Supplementary Table S3.

Supplementary Table S4.

Supplementary Table S5.
