# Supplementary material for: GWAS identifies an ortholog of the rice D11 gene as a candidate gene for grain size in an international collection of hexaploid wheat
Source: Sci Rep. 2021 Sep 30;11:19483. doi: 10.1038/s41598-021-98626-0 (PMC8484655; doi:10.1038/s41598-021-98626-0)
Supplement: Supplementary file 1 — Supplementary Figures. [file 41598_2021_98626_MOESM1_ESM.docx]

GWAS identifies an ortholog of the rice D11 gene as a candidate gene for grain size in an international collection of hexaploid wheat

### Honoré Tekeu^1,2,5^, **Eddy L.M. Ngonkeu^4,5^,** Sébastien Bélanger^2,3^**, Pierre F. Djocgou**é**^5^,** Amina Abed^1,2^**, Davoud Torkamaneh^1,2,^**^6^**, Brian Boyle^2^**, **Patrick M. Tsimi^5^**[, Wuletaw](https://www.researchgate.net/profile/Wuletaw_Tadesse2) Tadesse^7^, **Martine Jean^1,2^,** François Belzile^1,2^*

^1^Département de Phytologie, Université Laval, Quebec City, QC, Canada

^2^Institut de Biologie Intégrative et des Systèmes, Université Laval, Quebec City, QC, Canada

^3^Donald Danforth Plant Science Center, St.Louis, Missouri*,* USA

^4^Institute of Agricultural Research for Development, Yaoundé, Cameroon

^5^Department of Plant Biology, University of Yaoundé I, Yaoundé, Cameroon

^6^Department of Plant Agriculture, University of Guelph, Guelph, ON, Canada

^7^International Center for Agricultural Research in the Dry Areas (ICARDA), Beirut, Lebanon


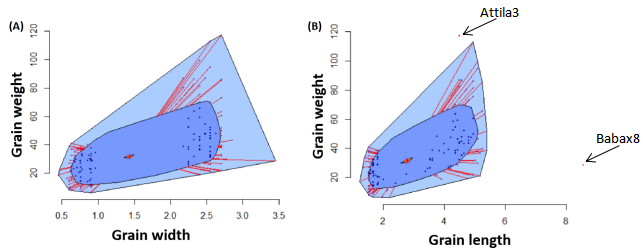


Figure S1. Bagplot showing the relationship between grain traits. Plots indicate that all accessions that there were no outliers when considering the relationship between grain weight and grain width (A) whereas two accessions (Attila3 and Babax8) were declared as outliers when comparing grain weight and length (B). In the case of Attila3, grain weight was exceptionally high given its length, while in the case of Babax8, the opposite was true.


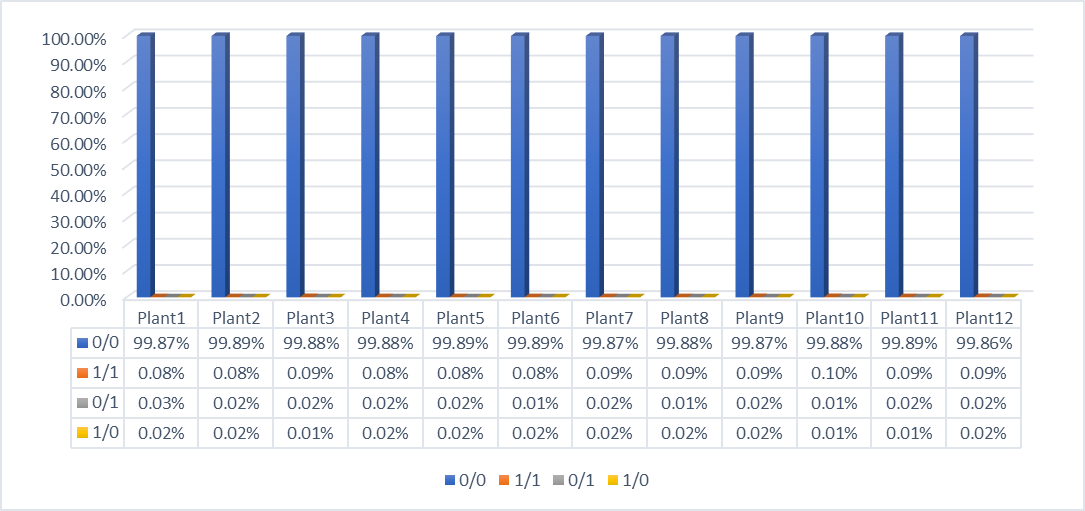


Figure S2: Accuracy of a non-imputed GBS dataset from 12 plants of Chinese Spring using a dataset of 129,940 SNPs. Imputed genotypes were compared to genotypes from the Chinese Spring reference genome. 0/0: Homozygous for the reference allele; 1/1: Homozygous for the alternative allele; 0/1 or 1/0: Heterozygous.


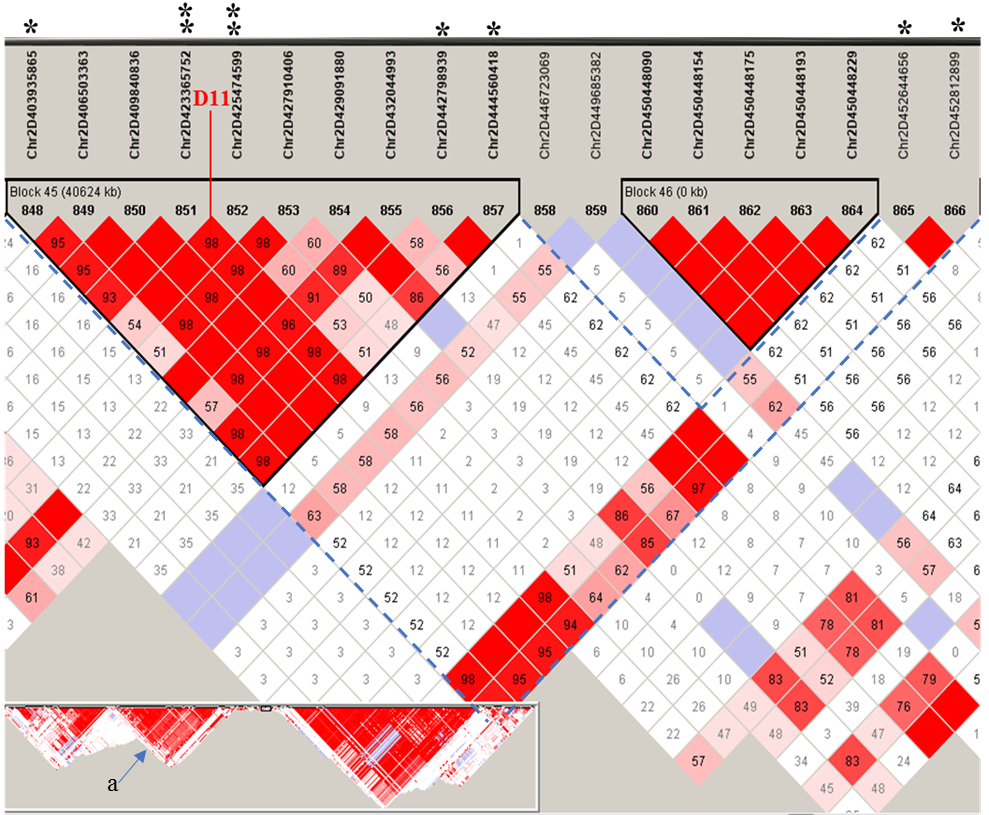


Figure S3: Linkage disequilibrium plots across wheat lines showing SNPs markers associated with grain traits in linkage blocks on chromosome 2D. The box (bottom left: a) in the figure illustrates the LD in an area of ~ 45Mb around the peak SNP, with enlargement of the region around the candidate gene D11.

*: significant SNP markers identified by GWAS

**: SNP markers identified using HaplotypeMiner tools.

LD plots was generated using Haploview version 4.2 (https://www.broadinstitute.org/haploview/haploview).


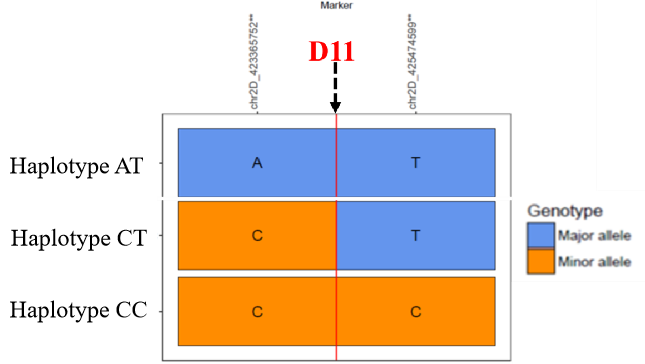


Figure S4: Three haplotypes identified around the D11 gene, using HaplotypeMiner version 0.1.0 (https://rdrr.io/github/malemay/HaplotypeMiner/)^.^


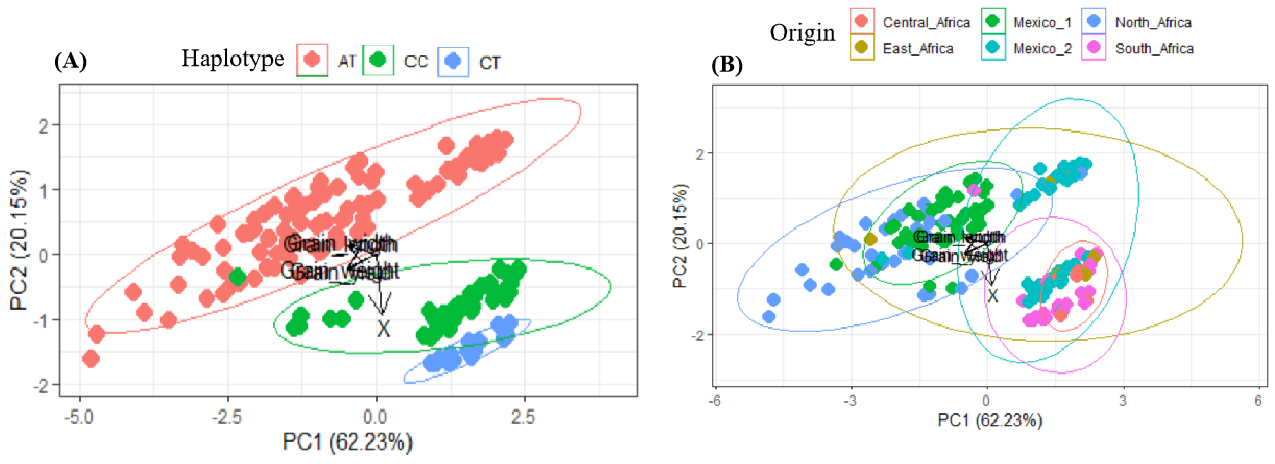


Figure S5: Relations between the 3 haplotypes (A) and the 6 groups found in the population analysis (B). The haplotype AT predominates in the populations of Mexico 1 and North Africa. Plots were generated using R version 4.1.0 (https://cran.r-project.org/bin/windows/base/).

Figure S6: Matrix of genomic relationships among wheat individuals, calculated using the Van Raden method in GAPIT version 2 (https://pubmed.ncbi.nlm.nih.gov/27898829/).
